# The efficacy and safety of pinocembrin in a sheep model of bleomycin-induced pulmonary fibrosis

**DOI:** 10.1371/journal.pone.0260719

**Published:** 2021-12-02

**Authors:** Habtamu B. Derseh, Jason Q. D. Goodger, Jean-Pierre Y. Scheerlinck, Chrishan S. Samuel, Ian E. Woodrow, Enzo A. Palombo, Alistair Cumming, Ken Snibson

**Affiliations:** 1 Melbourne Veterinary School, Faculty of Veterinary and Agricultural Sciences, University of Melbourne, Parkville, Victoria, Australia; 2 School of Biosciences, University of Melbourne, Parkville, Victoria, Australia; 3 Cardiovascular Disease Program, Monash Biomedicine Discovery Institute and Department of Pharmacology, Monash University, Clayton, Victoria, Australia; 4 School of Ecosystem and Forest Sciences, University of Melbourne, Parkville, Victoria, Australia; 5 Department of Chemistry and Biotechnology, Swinburne University of Technology, Hawthorn, Victoria, Australia; 6 Gretals Australia Pty Ltd, Mornington, Victoria, Australia; University of Michigan, UNITED STATES

## Abstract

The primary flavonoid, pinocembrin, is thought to have a variety of medical uses which relate to its reported anti-oxidant, anti-inflammatory, anti-microbial and anti-cancer properties. Some studies have reported that this flavonoid has anti-fibrotic activities. In this study, we investigated whether pinocembrin would impede fibrosis, dampen inflammation and improve lung function in a large animal model of pulmonary fibrosis. Fibrosis was induced in two localized lung segments in each of the 10 sheep participating in the study. This was achieved via two infusions of bleomycin delivered bronchoscopically at a two-week interval. Another lung segment in the same sheep was left untreated, and was used as a healthy control. The animals were kept for a little over 5 weeks after the final infusion of bleomycin. Pinocembrin, isolated from *Eucalyptus* leaves, was administered to one of the two bleomycin damaged lung segments at a dose of 7 mg. This dose was given once-weekly over 4-weeks, starting one week after the final bleomycin infusion. Lung compliance (as a measure of stiffness) was significantly improved after four weekly administrations of pinocembrin to bleomycin-damaged lung segments. There were significantly lower numbers of neutrophils and inflammatory cells in the bronchoalveolar lavage of bleomycin-infused lung segments that were treated with pinocembrin. Compared to bleomycin damaged lung segments without drug treatment, pinocembrin administration was associated with significantly lower numbers of immuno-positive CD8^+^ and CD4^+^ T cells in the lung parenchyma. Histopathology scoring data showed that pinocembrin treatment was associated with significant improvement in inflammation and overall pathology scores. Hydroxy proline analysis showed that the administration of pinocembrin did not reduce the increased collagen content that was induced by bleomycin in this model. Analyses of Masson’s Trichrome stained sections showed that pinocembrin treatment significantly reduced the connective tissue content in lung segments exposed to bleomycin when compared to bleomycin-infused lungs that did not receive pinocembrin. The striking anti-inflammatory and modest anti-fibrotic remodelling effects of pinocembrin administration were likely linked to the compound’s ability to improve lung pathology and functional compliance in this animal model of pulmonary fibrosis.

## Introduction

The primary flavonoid, pinocembrin, has been identified and isolated from a number of plant species. This bioactive molecule is thought to have a variety of medical uses which relate to its reported anti-oxidant, anti-inflammatory, anti-microbial, and anti-cancer properties [1–7]. Importantly, pinocembrin has been shown to have anti-fibrotic properties in a number of different animal models of disease. For example, pinocembrin lowers ventricular fibrosis [8] in a rat model of depression and lowers atrial fibrosis in a rat model of myocardiac infarction [9]. Similarly, in an experimental rat model of chloroform-induced liver fibrosis, oral administration of pinocembrin reduced collagen and alpha-SMA (used as a marker of myofibroblast differentiation) expression, as measures of fibrosis [10]. A recent study demonstrated Pinocembrin alleviates bleomycin-induced skin fibrosis in mice and suppressed proliferation, migration, and invasion of keloid fibroblasts and mouse primary dermal fibroblasts in vitro [11]. Pinocembrin induced these anti-fibrotic effects via the downregulation of the NF-κβ and TGF-β1/Smad signaling pathways [10, 11].

Given the known anti-fibrotic effects of pinocembrin, we aimed to ascertain whether pinocembrin would impede fibrosis and improve lung function in a large animal model of pulmonary fibrosis. The physiologically and pharmacologically relevant model for pulmonary fibrosis was developed as a vehicle for investigating the underlying disease mechanism(s) for the human condition idiopathic pulmonary fibrosis (IPF) [12, 13]. It has also been useful for testing new treatments against pulmonary fibrosis [14–16].

Idiopathic pulmonary fibrosis (IPF) is a devastating chronic lung disease characterized by progressive lung scarring due to exuberant accumulation of extracellular matrix (ECM) in the lung parenchyma [17]. The prognosis of IPF is very poor with only 3–5 years of median survival of patients after diagnosis [18]. Current available pharmacological treatments are limited to Pirfenidone and Nintedanib, drugs that have been shown to only slow the progression of the disease, without halting it. In addition, there are significant adverse side-effects associated with these two drugs. Therefore, there is an unmet need for new effective treatments for this condition.

The aim of this study was to evaluate the therapeutic efficacy and safety of pinocembrin in a sheep model of bleomycin-induced pulmonary fibrosis developed in our laboratory (12). Specifically, a study was performed to ascertain whether local lung administration of pinocembrin significantly attenuated fibrosis, inflammation and histopathology in specific lung-segments following induction of fibrosis with bleomycin. Importantly, we determined whether local administration of pinocembrin significantly improved the physiological function of specific lung-segments with bleomycin-induced fibrosis.

## Materials and methods

### Induction of fibrosis in sheep lung segments

In this study, we used a total of 10 sheep. We induced fibrosis in two lung segments of each sheep in a similar manner to our previously studies [12, 13]. Using this established procedure (see details below) the fibrosis was confined to small, isolated regions in the lungs leaving the remaining 90–95% of the healthy unaffected lungs to undertake normal respiratory function.

**Fig 1:**
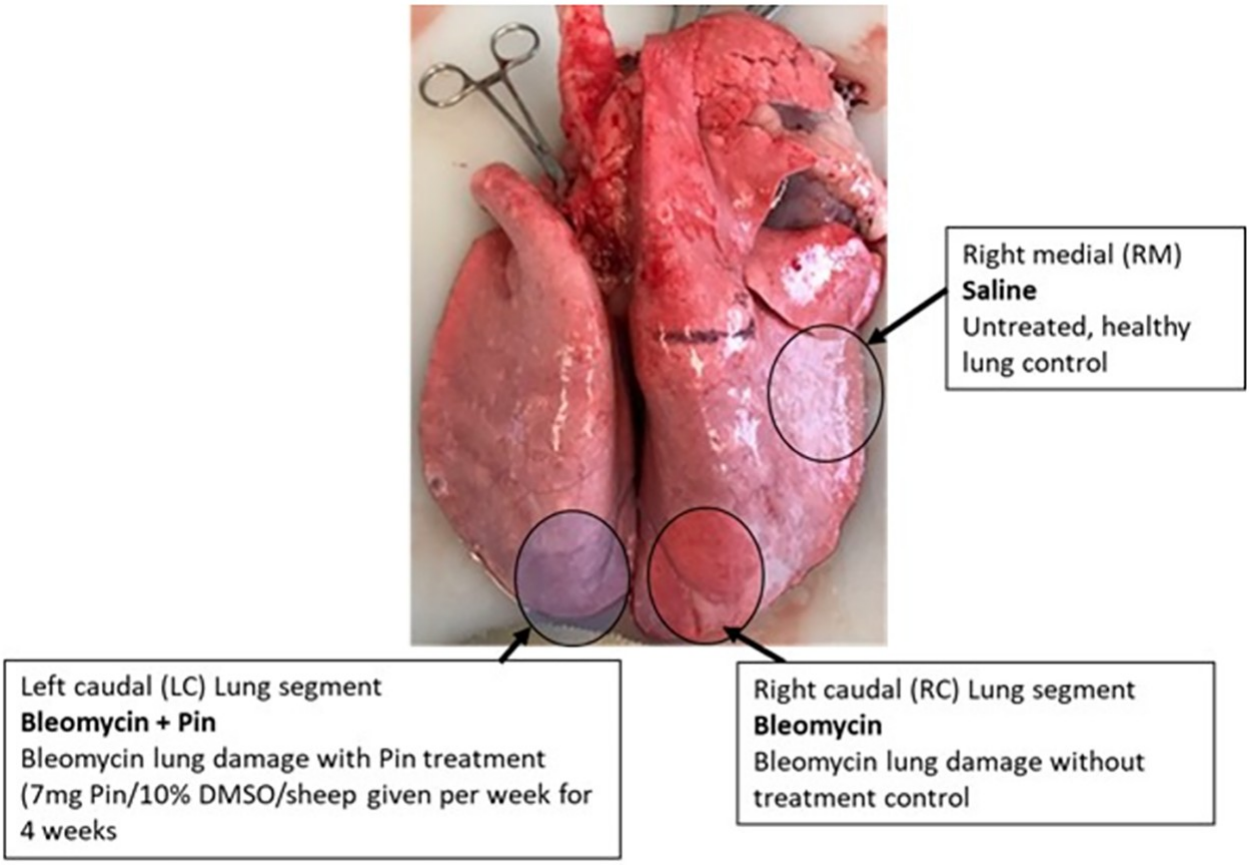
Experimental design. Locations of spatially separate lung-segments, which received the different treatments. Note that the treatments to either the left or right caudal segments were randomized, so that half (n = 5 of the sheep) received the treatments as shown above, while for the other (n = 5 sheep), the treatments in the caudal segments were reversed. Note that the rest of the lung was available for healthy respiration.

All sheep in the study were challenged with 2 single doses of bleomycin (3 units) per lung segment, 2 weeks apart and the animals were kept for a little over 5 weeks after the final bleomycin dose The administration procedure involved inserting a bronchoscope into lung segments in the right and left lung, and then slowly infusing via the bronchoscope biopsy port, the bleomycin into two segments as shown in Fig 1.

**Fig 2:**
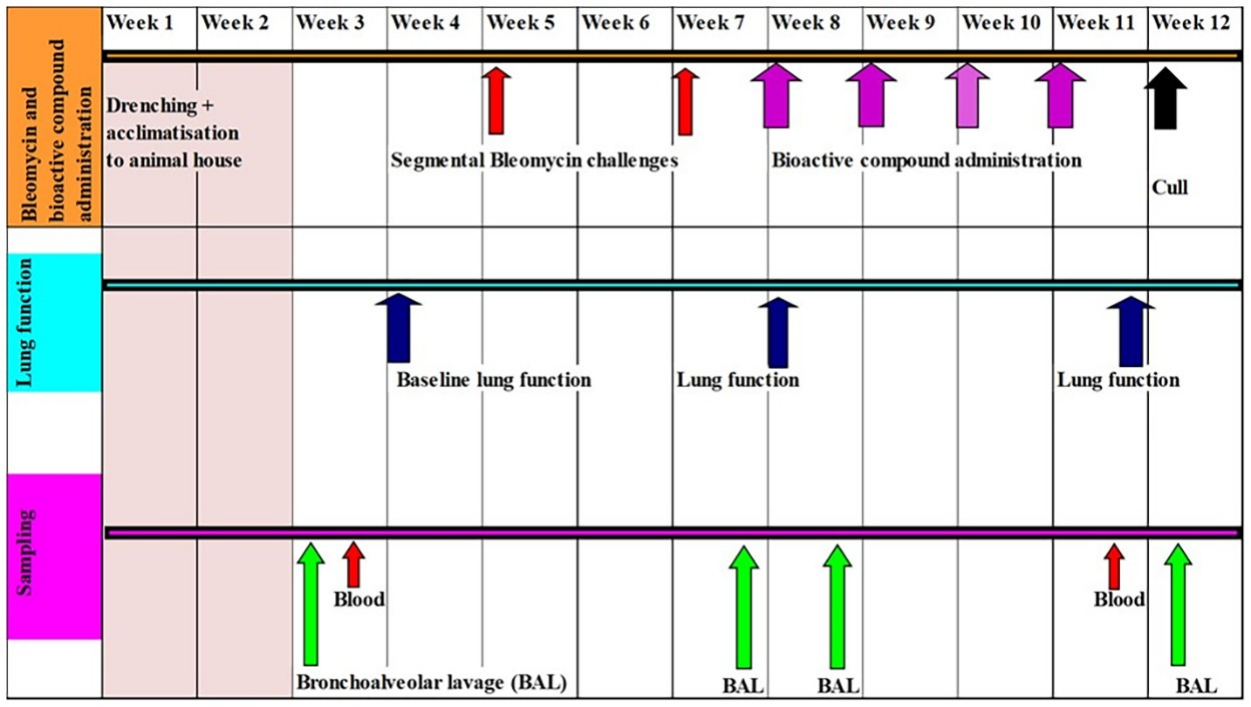
Study timeline for bleomycin challenges, bioactive compound treatments, lung function assessments, and sampling time.

The timing of bleomycin infusions, blood sampling, bronchoalveolar lavage sampling and lung function testing and application of pinocembrin are detailed in Fig 2. All experimental animal procedures were approved by the University of Melbourne animal ethics committee (animal ethics approval ID number: 2015209.1). These experimental procedures and timelines associated with the animal work were designed to alleviate and minimize suffering of the sheep used in the study. In particular, the number of bronchoscopic procedures were reduced to the bare minimum that would allow for successful interpretation of the data.

### Isolation and structural confirmation of pinocembrin

Pinocembrin was purified from methanol extracts of leaves collected from three-year-old *Eucalyptus pressiana* subsp. *pressiana* saplings were grown in an experimental plot at the University of Melbourne Dookie campus (36°23’3”S, 145°42’52”E). Seed purchased from Nindethana Seed Company (King River, Australia) had been collected from Woodenellup, Western Australia and seedlings were raised and transplanted to the plot as described in [19]. Sampled leaves were dried in an oven at 50°C for 48 h before being ground to a fine powder using an IKA MF10 mill fitted with a 1 mm mesh. Ground tissue (100 g) was placed in Kinesis KX cellulose thimbles (Antylia Scientific, Chicago, USA) and subjected to Soxhlet extraction with 100% MeOH (200 mL) for 8 h. MeOH extracts were then concentrated to approximately 50 mL using rotary evaporation. Aliquots of concentrated extracts (2 mL) were subjected to Flash chromatography on a Reveleris X2 system (Buchi AG, Flawil, Switzerland) fitted with a FlashPure C18 RP-WP 12 g cartridge (Buchi). The solvent system used was a gradient of acetonitrile (MeCN) and water from 30–100% MeCN over 5 min with a 1 min equilibration time (S1 Fig). The pinocembrin-containing fraction was detected at 283 nm and collected. Fractions from multiple Flash runs were pooled, the MeCN removed via rotary evaporation and the pinocembrin dried using lyophilization. The structure of pinocembrin was confirmed via ESI-LCMS and NMR spectroscopy as described in [19] with comparison to a commercial standard (Sigma-Aldrich, St. Louis, USA). Pinocembrin: Pale yellow crystals from MeCN/H_2_O. UV λ_max_ MeCN (nm): band-I 325 nm, band-II 289 nm. ^1^H NMR (600 MHz, CDCl_3_): δ 5.36 (1H, dd, J 13.0, 3.0 Hz, H-2), δ 2.76 (1H, dd, J 17.1, 3.1 Hz, H-3a), δ 3.02 (1H, dd, J 17.1, 13.1 Hz, H-3b), δ 5.92 (1H, d, 2.2 Hz, H-6), δ 5.94 (1H, d, 2.2 Hz, H-8). ESI-MS (fragmentor 150V, Skimmer 65V) *m*/*z* (rel. int. %): 255.0673 ([M-H]^-^, 100), 213.0565 ([M-42-H]^-^, 19), 151.0037 ([M-104-H]^-^, 16), 257.0811 ([M+H]^+^, 100), 153.0167 ([M-104+H]^+^, 2). Mass-based purity of the isolated pinocembrin was quantified at a minimum of 96% using HPLC-PDA by comparison to a commercial standard (S2A and S2B Fig).

### Administration of pinocembrin to sheep lung segments

For each week of the dosing regime, 7 mg pinocembrin was infused into a single caudal lung segment in each sheep undergoing the trial (Fig 1). This dose weight of pinocembrin was based on a previously published study which showed that 50 mg pinocembrin per kg bodyweight was the most efficacious dose for ameliorating disease symptoms in that animal model [20]. The 7 mg pinocembrin dose allowed for the fact that only approximately 150gms of lung segment per sheep was treated at ~50 mg/kg. The bioactive was dissolved in 10% DMSO, and given once-weekly over a four-week period, as shown in Fig 2. For control purposes, the lung segment in the opposite lung was used as a bleomycin-positive, without drug, control, as indicated in Fig 1. The bioactive molecules in vehicle were delivered as 5 mL infusions through the biopsy port of the bronchoscope. Note that to nullify any small differences (e.g., physiological or anatomical etc.) between the left and right lungs, the infusions of pinocembrin were randomized between the left and right caudal lung segments. Half the animals (n = 5 sheep) received pinocembrin to the right caudal lung segments, while the other (n = 5 sheep) received pinocembrin to the left caudal lung segments. The right medial lung segment was left untreated and used for healthy lung control tissue which was sampled at autopsy (Fig 1).

### Bronchoalveolar lavage sampling procedures

For each of the bronchoalveolar (BAL) samplings at the timepoints listed in Fig 2, the endoscope was manipulated into a specific lung-segment for sampling, usually passing through about 3 to 4 airway branches. For BAL sampling, 10 mL of sterile saline was infused through the biopsy port of the endoscope into the specific lung-segment and then recovered into a syringe through the same port. This procedure recovered between 3 and 5 mL of BAL fluid. The sampling method collected cells for analyses from the small airway and alveolar lumens of the specific lung-segment where the bronchoscope was navigated to. The BAL cells from each segment were centrifuged onto glass slides and stained for differential cell counting of inflammatory cells with Hem-Quik, as previously described [13].

### Lung function testing and analyses

Lung function was measured in the described lung-segments at the time points indicated in Fig 2. Lung function was assessed in all test lung-segments (left and right caudal lobes, and medial lobe in each sheep). The functional capacity of the lung segments was measured through the endoscope using a published procedure [12, 13]. The lung function parameter assessed in this study was referred to as compliance in the lung segment (abbreviated to Cseg). In general, compliance is a measure of how easily it is to inflate the lung. A poorly compliant lung is referred to as a stiff lung, which is typically more difficult to inflate.

### Blood sampling

Whole blood was collected at baseline before induction of fibrosis and at the end of the experiment before euthanasia of experimental animals as indicated in Fig 2 by drawing blood from the jugular vein into a tube containing heparin. The blood was processed for blood cell count analysis.

### Lung tissue isolation and analyses

Sheep were euthanized a little over 5 weeks after the last bleomycin dose (early Week 12), as indicated in Fig 2. Euthanasia was induced via a barbiturate overdose given IV at 1ml per 2 Kg body weight as recommended by the manufacturer (Lethabarb, Virbac Animal health Pty Ltd). Following euthanasia, the lungs were removed, and targeted lung-segments identified and carefully dissected free from surrounding tissue. Individual segments were then inflated with a 1:1 mixture of OCT (Optimal Cutting Temperature, Tissue-Tek) and sterile PBS solution, under pressure of approximately 20 cm/H_2_O [13]. Several serial transverse sections of the inflated segment were fixed in 10% neutral-buffered formalin and processed in paraffin for histopathological assessment. The remaining lung slices were embedded in OCT and frozen in cryo-moulds on aluminium boats floating on liquid nitrogen.

Paraffin-embedded tissue sections (5 μm) were stained with haematoxylin and eosin Y (H&E) for general histology and with Masson’s trichrome staining to identify changes to collagen content within the lung parenchyma.

### Immunohistochemistry

Immunohistochemical localization of CD4^+^ and CD8^+^ T lymphocytes was performed on frozen sections. Lung tissue sections (5 μm) were fixed with ice cold ethanol; endogenous peroxides were blocked using H_2_O_2_ solution and sections were then incubated with primary antibodies (monoclonal mouse anti CD4 (neat) and monoclonal mouse anti CD8 (neat)) in humid chamber for 2 hours at room temperature. After incubation with primary antibodies, sections were then washed with PBS three times and incubated with secondary antibody (rabbit antimouse IgG (HRP) (ab6728)) (1:100; Abcam, Melbourne, Australia) for 1 hour at room temperature. To check specificity, isotype controls were included during IHC run using a biologically irrelevant SBU-3 antibody of the same isotype. The sections were counter-stained with Mayer’s hematoxylin solution for 20 seconds. Antigen-antibody complex was visualized with 3,3′-diaminobenzidine. Finally, sections were dehydrated through ethanol, cleared in xylene and mounted in DPX.

### Histopathology scoring

Histopathology of the lung parenchyma was assessed using a semi-quantitative scoring system as previously described [12, 13]. Briefly, histology slides were all blinded so that the assessor did not know the treatment groups. For each H&E-stained section, 10 consecutive, non-overlapping fields at x200 magnification were graded based on the scoring criteria for fibrosis, inflammation and overall pathology scores as previously reported [12, 13]. The areas were selected away from large airways and major blood vessels. Scores from all ten fields were then averaged to give representative scores for the parameters assessed in the sectioned lung segment.

### Analysis of collagen protein concentration using the hydroxyproline assay

The hydroxyproline assay was used to extrapolate the collagen content and concentration of each segment, as described previously (4,5). Briefly, frozen lung tissues from each segment were lyophilized to dry weight, hydrolyzed in 6M HCl, and assessed for hydroxyproline content by measuring the absorbance of reconstituted samples (in 0.1M HCl) at 558 nm using a Beckman DU-64 spectrophotometer (Beckman Coulter Inc, Brea, CA). Hydroxyproline content was determined from a standard curve of trans-L-hydroxy-L- proline (Sigma-Aldrich). Collagen content was extrapolated by multiplying the hydroxyproline measurements by 6.94 (based on hydroxyproline representing ~14.4% of the amino acid composition of collagen in most tissues [21]) and then expressed as a proportion of the dry weight tissue to yield collagen concentration (which was expressed as a percentage).

### Analysis of connective tissue content for assessing the degree of fibrosis

The degree of fibrosis was quantified by assessing the changes in overall connective tissue content within the parenchymal tissue using a previously described method [13]. To perform this analysis, paraffin sections of sheep lung tissues were stained using a Masson’s trichrome stain which stains connective tissue blue. Subsequently, images of Masson’s trichrome-stained lung section were captured using a digital camera linked to microscope and computer. Ten fields were randomly captured under x400 magnification excluding large blood vessels and bronchi. The images were then analyzed using Image-Pro^®^ Plus (Version 6.3 for Windows, Media Cybernetics, Bethseda, Maryland, USA) using the ‘colour selector’ tool to measure the area of blue-stained tissues (collagen and other connective tissues) within each field of view. The values for each of the ten images were then averaged for each slide. The fraction of blue stained tissue area was expressed as a percentage per total field area (percentage of blue stained tissue area per total field area). Image capturing and analysis were performed in a blinded manner on coded slides.

### Statistical analysis

Statistical analysis was performed using GraphPad Prism for Windows Version 5.01 (GraphPad Software Inc., La Jolla, CA, United States). Parameters were assessed for Gaussian distribution using the D’Agostino normality and Pearson omnibus test. For parametric data, paired two-tailed t-tests were performed to compare between lung segments received bleomycin only with saline-infused control segments and lung segments received bleomycin plus pinocembrin infusion. For data that did not meet assumptions for Gaussian distribution, the Wilcoxon matched-pairs signed rank test was used. In all cases, a p-value less than 0.05 was defined as a significant difference. All values are reported as means (± SEM).

## Results

### Animal health and the safety of pinocembrin administration

Health checks were routinely performed throughout the treatment periods and trial until euthanasia. This was to ascertain that pinocembrin treatment caused no untoward health issues, or adverse side-effects, to the sheep undergoing the trial. All animals remained healthy throughout the pinocembrin treatment period (week 8 to week 12, Fig 2). During this period the animals continued to gain weight in the expected normal range for these sheep, had normal white blood cell counts, and there were no otherwise adverse health events.

### Pinocembrin administration improves the lung function decline associated with the development of bleomycin-induced pulmonary fibrosis

**Fig 3:**
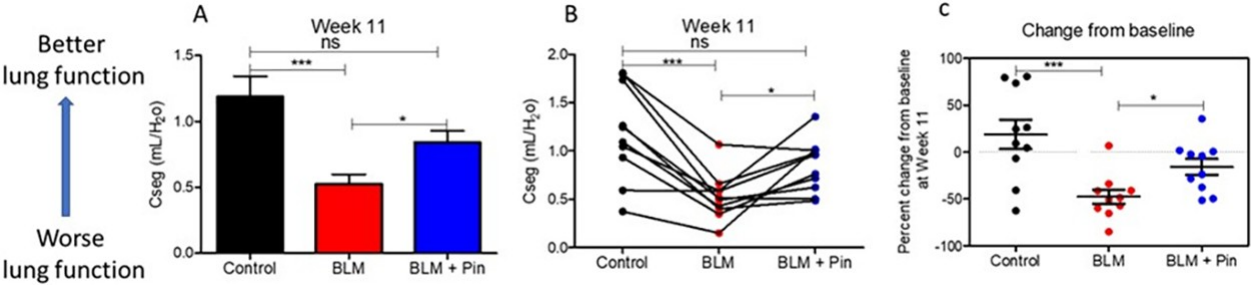
Lung function in the differentially treated lung segments as assessed at week 11 of the study. The differentially treated lung segments were the right medial (RM) lung-segments which were left untreated for healthy lung controls (Control), the right caudal (RC) and the left caudal (LC) lung-segments which were either infused with bleomycin without drug treatment (BLM), or infused with bleomycin and received 4 once-weekly doses of pinocembrin (BLM + PIN). Part A shows mean data for Cseg (n = 10), which is a measure for how easy it is to inflate the lung segment. Part B shows individual sheep data. Part C shows percent change of Cseg at week 11 from baseline values taken at week 0 at the beginning of the study. Significance was determined using paired t-tests, *p<0.05, ***p<0.001, n = 10 sheep.

Fig 3 shows lung function of the different lung-segments after once-weekly treatments with pinocembrin for four weeks. The lung function parameter measured was compliance in local lung segments and is referred to as segmental compliance (Cseg). Generally, lower levels of compliance mean poorer function in the lung-segment (i.e. more difficult to inflate and the lung is stiffer). As expected, the lung-segments which received the damaging agent bleomycin alone, without pinocembrin, had significantly lower mean segmental compliance than the untreated healthy control lung-segments (Fig 3A). The lung-segments, which received the damaging agent bleomycin with pinocembrin administration, had higher mean segmental compliance, which was not significantly different from the untreated healthy control lung segments (Fig 3A). Another lung function assessment used was the percentage change in compliance from baseline (Fig 3C). This measured the change in compliance from the start of the study (before bleomycin and pinocembrin treatments) to after the completion of the final pinocembrin treatment. The assessment showed that compliance in pinocembrin-treated lung segments had significantly improved after the once-weekly administrations of pinocembrin over the 4-week treatment period (Fig 3C).

### Pinocembrin administration reduces bleomycin-induced inflammatory cell accumulation in BAL fluid

**Fig 4:**
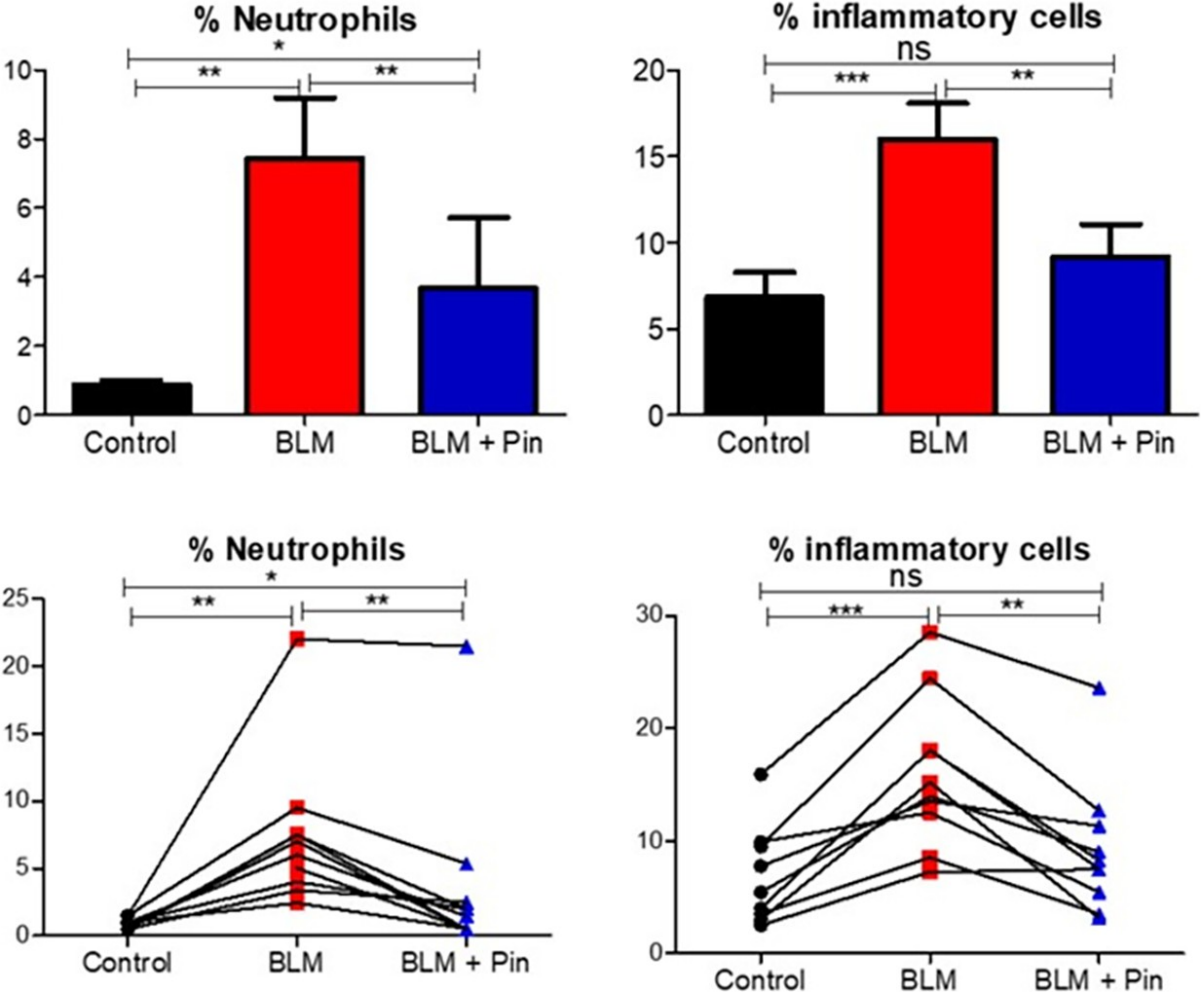
Neutrophils and inflammatory cells recovered from the bronchoalveolar lavage (BAL) fluid of the differentially treated lung-segments at week 12. The differentially treated lung segments were the right medial (RM) lung-segments which were left untreated for healthy lung controls (Control), the right caudal (RC) and the left caudal (LC) lung-segments which were either infused with bleomycin without drug treatment (BLM) or infused with bleomycin and received 4 once-weekly doses of pinocembrin (BLM + PIN). The left panels show neutrophil data, and the right panels show inflammatory cell data, which included the sum of the percentages of neutrophils, lymphocytes, and eosinophils. The top panels show mean data for ten sheep. The bottom panel shows individual sheep data. Significance was determined using paired t-tests, *p<0.05, **p<0.01, ***p<0.001, n = 10 sheep.

Fig 4 shows BAL cell data after once-weekly infusion treatments with pinocembrin over a 4-week treatment period. The BAL cells were sampled from lung-segments during week 12 of the trial, two days before the sheep were culled. The cell counts assessed in the BAL fluid were neutrophils alone, and the sum of the main inflammatory cells, which included the neutrophils, eosinophils and lymphocytes. As expected, the healthy control lung segments, which were untreated, had relatively low numbers of inflammatory cells in the BAL fluid (Fig 4).

In contrast, the lung-segments injured by bleomycin, without pinocembrin, had significantly high numbers of neutrophils and other inflammatory cells in the BAL fluid compared to healthy control segments (Fig 4). The lung-segments which received the damaging agent bleomycin, and had pinocembrin treatments, showed significantly lower numbers of neutrophils and inflammatory cells when compared to the cell numbers sampled from lung-segments, which received bleomycin alone without pinocembrin (Fig 4).

### The effect of pinocembrin on lung parenchymal T cells

**Fig 5:**
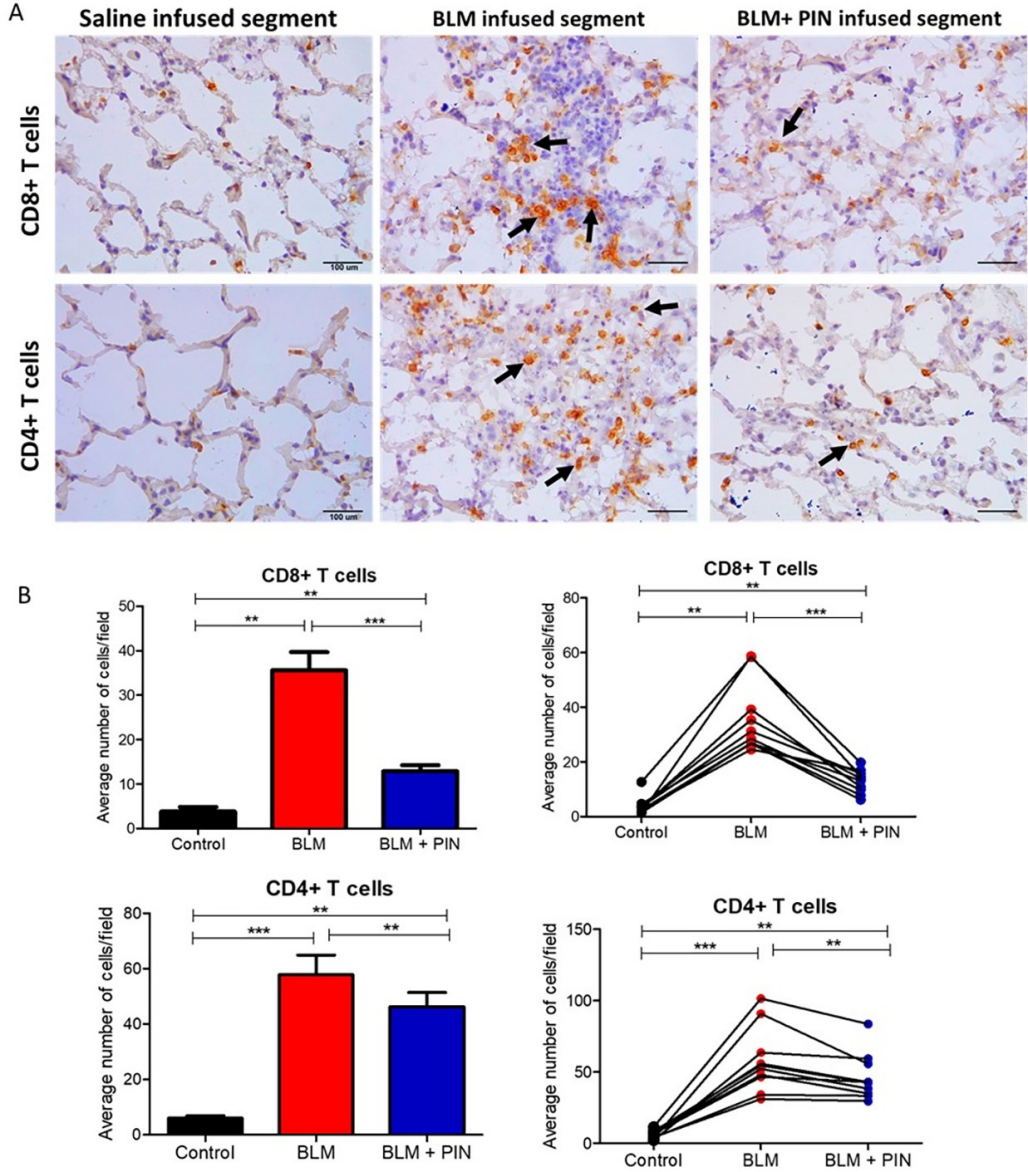
The effect of pinocembrin on lung parenchymal T cells. (A) representative photomicrographs showing CD8^+^ and CD4^+^ T cells (arrows show examples of immuno-positive cells) in the lung parenchyma sampled from the differentially treated lung-segments at week 12. (B) graphs showing the average number of positive cells per field at 400x magnification for each differentially treated segment. The differentially treated lung segments were the right medial (RM) lung-segments which were left untreated for healthy lung controls (Control), the right caudal (RC) and the left caudal (LC) lung-segments which were either infused with bleomycin without drug treatment (BLM) or infused with bleomycin and received 4 once-weekly doses of pinocembrin (BLM + PIN). The left panels show mean lung segment data and the right panels show individual sheep data. Significance was determined using paired t-tests, **p<0.01, ***p<0.001, n = 10 sheep. Scale bars = 100 μm.

Fig 5 shows T cell data after four infusion treatments with pinocembrin, administered once weekly. T cells were assessed in the parenchyma of the differentially treated lung-segments, which were sampled at post-mortem (week 12). As expected, the healthy control lung segments, which were untreated, had relatively low numbers of CD8^+^ and CD4^+^ T cells in the lung parenchyma (Fig 5).

In contrast, the lung-segments injured by bleomycin, without pinocembrin treatment, had significantly higher numbers of CD8^+^ and CD4^+^ T cells in the lung parenchyma compared to healthy control segments (Fig 5). The lung-segments which received the damaging agent bleomycin, and had pinocembrin treatments, showed significantly lower numbers of CD8^+^ and CD4^+^ T cells in the lung parenchyma when compared to the cell numbers sampled from lung-segments, which received bleomycin alone without pinocembrin (Fig 5).

### The effects of pinocembrin on histopathology

**Fig 6:**
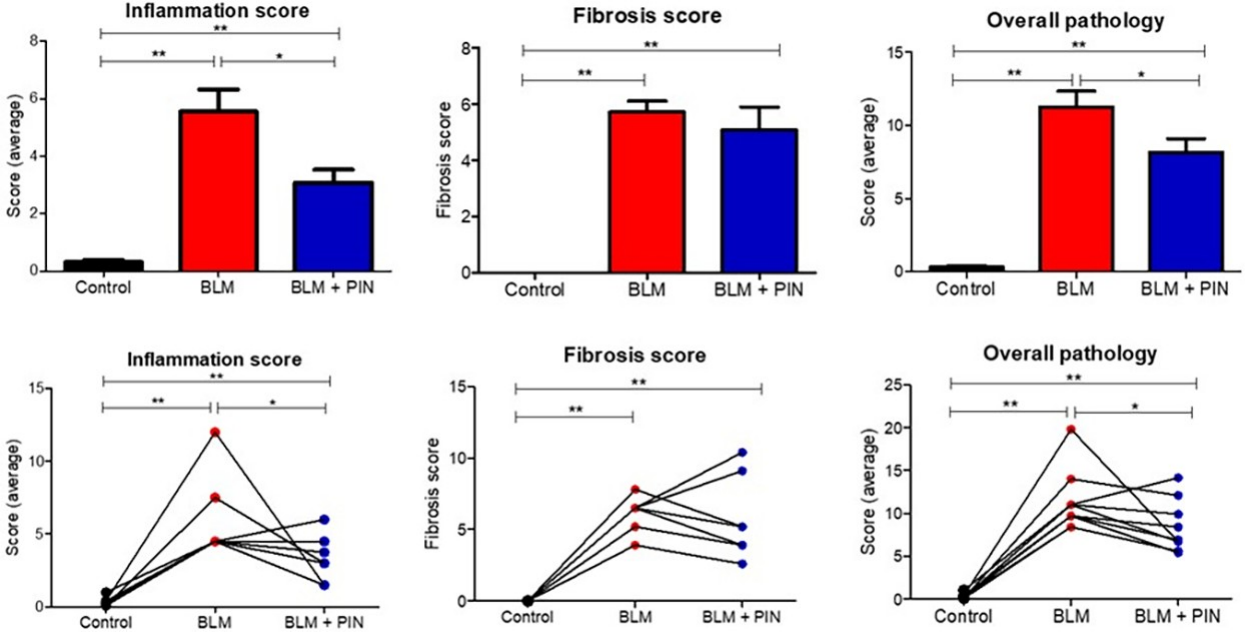
Histopathology scoring data as assessed on histological H+E-stained sections sampled at post-mortem from the differentially treated lung-segments. The differentially treated lung segments were the right medial (RM) lung-segments which were left untreated for healthy lung controls (Control), the right caudal (RC) and the left caudal (LC) lung-segments which were either infused with bleomycin without drug treatment (BLM) or infused with bleomycin and received 4 once-weekly doses of pinocembrin (BLM + PIN). The top panels show mean scoring data for ten sheep. The bottom panels show individual sheep data. Significance was determined using paired t-tests, *p<0.05, **p<0.01, n = 10 sheep. Scoring criteria is described in the Materials and methods.

Fig 6 shows histopathology scoring data after four once-weekly treatments with pinocembrin. The histopathology parameters scored were inflammation, fibrosis and overall pathology. As expected for a normal healthy lung, the control lung-segments which were left untreated, had low scores for each pathology parameter assessed (Fig 6). In contrast, the lung-segments injured by bleomycin, without pinocembrin treatment, had significantly high mean scores for each parameter tested (Fig 6). Importantly, the lung-segments, which received the injuring agent bleomycin, and had pinocembrin treatments, had lower mean scores for each parameter assessed (Fig 6). Pinocembrin treatment significantly reduced inflammation and overall pathology scores compared to segments which received bleomycin infusion only (Fig 6). While the lung segments which received bleomycin and pinocembrin infusion had lower fibrosis scores as compared to segments which received bleomycin infusion only, the difference was not statistically significant (Fig 6).

### The effects of pinocembrin on connective tissue content

**Fig 7:**
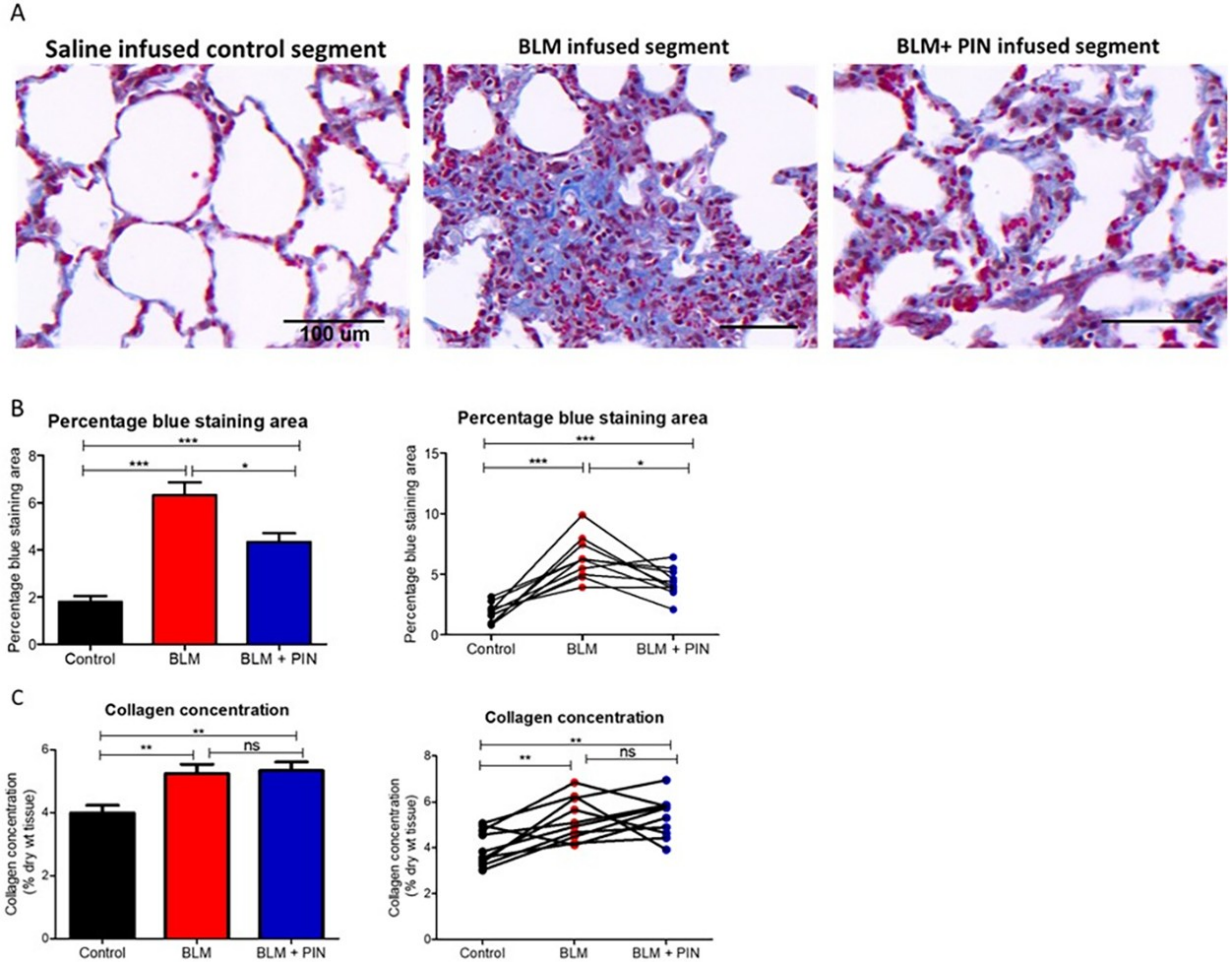
The effects of pinocembrin on connective tissue content. (A) Shows representative photomicrographs of Masson’s trichrome stained lung parenchyma from the differentially treated lung-segments. Masson’s Trichrome stains most connective tissues blue. The stained connective tissue includes collagen and other extracellular matrix proteins associated with fibrosis. The scale bar in each image indicates a length of 100 μm. (B) shows data for percentage of Masson’s trichrome stained lung tissue sections. (C) shows data for the hydroxyproline assay to determine collagen content after four once-weekly treatments with pinocembrin. For each sheep, the differentially treated lung segments were the right medial (RM) lung-segments which were left untreated for healthy lung controls (Control), the right caudal (RC) and the left caudal (LC) lung-segments which were either infused with bleomycin without drug treatment (BLM) or infused with bleomycin and received 4 once-weekly doses of pinocembrin (BLM + PIN). In the graphs B and C, the left panels show mean data. The right panels show individual sheep data. Significance was determined using paired t-tests, *p<0.05, **p<0.01, ***p<0.001, n = 10 sheep.

Microscopic observations of Masson’s trichrome stained sections showed that pinocembrin treatment reduced the amount of connective tissue that was induced by bleomycin infusion (Fig 7A). Fig 7B shows data for percentage of Masson’s Trichrome stained connective tissue area after four once-weekly treatments with pinocembrin. The data in Fig 7B showed that pinocembrin treatment significantly reduced the percentage blue staining in lung segments exposed to bleomycin when compared to bleomycin-infused lung which did not receive pinocembrin treatment (Fig 7B). Fig 7C shows data for the hydroxyproline assay for collagen content after four weekly treatments with pinocembrin. The data in Fig 7C were collected from all 10 animals in the trial and showed that bleomycin infusion alone (without pinocembrin) significantly increased collagen protein concentration compared with respective measurements from healthy lung control segments, which did not receive either bleomycin or pinocembrin. The administration of pinocembrin, however, did not reduce the bleomycin-induced increase in collagen concentration (Fig 7C).

## Discussion

This study made use of a physiologically and pharmacologically relevant sheep model for pulmonary fibrosis [12, 13] to ascertain the efficacy of pinocembrin. Overall, we found that pinocembrin treatment was efficacious in ameliorating the experimentally-induced lung pathology and caused no untoward health effects in all ten sheep undergoing the trial.

### The efficacy of pinocembrin to ameliorate several disease parameters in a sheep model of experimental lung disease

In the current study, we found that pinocembrin was able to ameliorate several disease parameters in the sheep model of pulmonary fibrosis. Our main findings were that the administration of pinocembrin was able to improve lung function, attenuate lung inflammation, and decrease the overall pathology scores, which were induced by bleomycin injury. Importantly, the statistical analyses of the data revealed that these disease readouts were significantly improved in pinocembrin-treated lung segments when compared with the corresponding data from bleomycin-injured and untreated lung segments.

In the sheep model of bleomycin-induced pulmonary fibrosis, bleomycin injury causes loss of localized lung compliance (a measure of lung stiffness) in bleomycin infused lung segments [12–14, 16, 22, 23]. In the current study, we found that pinocembrin treatment significantly improved functional compliance in the lung segments injured by bleomycin. This demonstrated that pinocembrin treatment in injured lung segments resulted in those segments functioning at higher levels than if left untreated.

In the current study, the identification of lung lavage inflammatory cells recovered from lung segments showed that pinocembrin treatment significantly reduced the number of inflammatory cells that occupy the luminal spaces of alveoli and small airways. The main inflammatory cell type that was reduced in the lung lavage fluid was neutrophils, which dropped from 7.4% of total BAL cells in the bleomycin without drug treatment lung segments to 3.7% in the pinocembrin-treated bleomycin-injured lung segments. The pinocembrin-associated reduction in CD4^+^ and CD8^+^ T cells in the lung parenchyma was entirely consistent with the reduction of inflammatory cells recovered from the lung lavage fluid of pinocembrin- and bleomycin-exposed lung segments. CD4^+^ and CD8^+^ T cells are important components in the cellular arms of many immune responses. Overall, these data support the notion that pinocembrin had strong anti-inflammatory properties in the disease model studied.

In terms of histopathology scores, the mean inflammation and overall pathology scores were improved in the injured lung segments after pinocembrin treatment. Importantly, these readouts, were statistically lower in the pinocembrin-treated damaged lungs, compared to the experimentally injured lungs without pinocembrin treatment. While the mean fibrosis pathology scores were lower in the pinocembrin-treated damaged lungs, compared to the experimentally injured lung without pinocembrin treatment, these did not reach statistical significance.

As the fibrosis pathology score data were not definitive for pinocembrin, the hydroxyproline assay was performed on tissue samples from the differentially treated lung segments. The hydroxyproline assay measures the level of collagen in a protein sample and is a gold standard readout measure for quantitating the levels of fibrosis in tissues. This assay showed that pinocembrin did not reduce the deposition of extracellular collagen associated with bleomycin injury. The Masson’s trichrome assay (another readout measure that is frequently used to assess the extent of fibrosis) showed that the percentage blue staining (i.e. a measure of connective tissue content) was significantly lower in bleomycin exposed and pinocembrin treated lung sections, when compared to bleomycin exposed lung sections which did not receive drug treatments. Taken together, data from both the hydroxyproline and Masson’s trichrome assays suggested that pinocembrin had the ability to reduce some extracellular matrix proteins (shown by Masson’s trichrome data), but not necessarily collagen (as corroborated by the hydroxyproline data). Overall, the data from fibrosis scores, Masson’s trichrome and hydroxyproline assays suggested that pinocembrin had only modest anti-fibrotic and anti-remodelling properties.

It had been previously shown that many bioactive compounds have anti-inflammatory as well as anti-fibrotic properties through modulation of several profibrotic and proinflammatory biomarkers such as TNFα, TGF-β, endothelin-1, IFN-γ, interleukins, VEGF, FGF-2, PDGF MMP-9, TIMPs, NF-kB, and collagen-I [24]. Furthermore, it had been found that the administration of anti-inflammatory compounds to bleomycin-infused mouse models of pulmonary fibrosis often resulted in a reduction of the fibrotic pathology. This was especially so if the anti-inflammatory molecules were applied early on after bleomycin exposure during the inflammatory (pre-fibrotic) stage of the pathology in these models [25]. Importantly though, when many of these anti-inflammatory molecules were tested in the clinic to treat fibrotic conditions, they were found to be ineffective [25]. In animal models where pinocembrin had been found to effectively retard fibrosis that was induced in other organs, pinocembrin also inhibited pro-inflammatory cytokines TNF-α, IL-1β and IL-6 and dampened inflammatory responses via depression of the NF-κβ signalling pathway [9, 10]. Further mechanistic studies with mouse LPS and bleomycin models suggested that TLR4 (toll-like receptor 4) and inflammasomes are causally linked to the pinocembrin suppression of NF-κβ inflammatory signalling [2]. As with all bleomycin-induced models of pulmonary fibrosis, the timing of the drug intervention is crucial for determining the relative anti-fibrotic and anti-inflammatory contributions of the treatment [25]. The timing of the pinocembrin treatment in the current study was 7 days after the final bleomycin infusion which is at the start of the fibrotic phase in this model [12, 13]. Although the inflammation was much lower at this day-7 timepoint, it was still nevertheless present, thus we cannot rule out that some of the weak anti-fibrotic effects of pinocembrin were due to its anti-inflammatory activities.

Previously, we have utilized this sheep model of pulmonary fibrosis to evaluate the therapeutic potential of two other drugs, the results of which may be useful for comparing the effects of pinocembrin found in the current study. We have tested an inhibitor of the KCa3.1 ion channel called senicapoc [15, 16], and we also investigated the efficacy of the FDA-approved drug pirfenidone in the model [14]. The published study designs were essentially similar to the current pinocembrin study, with each study using 10 sheep per group. The drug treatment periods were of a similar duration, being four to five weeks long for each drug. The main difference being that in the senicapoc and pirfenidone studies, the drugs were administered orally, while pinocembrin was administered intra-lung to local lung segments. Moreover, the whole lung did not receive pinocembrin in the current study, whereas the whole lung was exposed to drug in the pirfenidone and senicapoc trials. Analyses of the data between these studies reveals that the levels of improvements in disease markers of lung compliance, overall pathology scores and inflammation were at a similar level for the pinocembrin trial when compared to the other drug trials conducted with the sheep model. The one major point of difference between these studies was that in the previously published studies, the administration of pirfenidone or senicapoc resulted in significant reductions in collagen protein concentration, as assessed by the hydroxyproline assay [14, 16] Overall, the comparison between the trialed drugs suggests that the significant improvement in function and pathology of the lung is predominantly via the anti-inflammatory effects of pinocembrin, and to a lesser extent due to its anti-fibrotic actions.

In the current study, the four 7 mg doses of pinocembrin administered to the lungs at weekly intervals was found to be well-tolerated and were without any adverse side effects. This is consistent with data from a randomised double-blind, placebo-controlled trial with 58 healthy patients which showed that intravenous injection of pinocembrin was well tolerated up to 120 mg per day [26]. The trial assessed both single and multiple dose regimes and found that there were no major safety concerns that would preclude any future clinical development of the administration of pinocembrin via injection [26]. Data in the current study showed that the direct intra-lung delivery of pinocembrin was not only safe and viable, but also supported the notion that this flavonoid might be useful for treating a number of inflammatory diseases of the lung. Asthma is an important allergic airway disease that would be amenable to pinocembrin delivery to the lungs. Preliminary evidence supporting the anti-asthmatic effects of pinocembrin have been shown in a study where intraperitoneal injections of pinocembrin inhibited allergic airway disease in mice [20]. It could, thus, be reasonably expected that the localized delivery of pinocembrin solely to lungs would be more efficacious in treating asthma-like conditions in the clinic.

## Conclusion

In conclusion, the strong and significant data derived from this study was consistent with pinocembrin being bioactive and improving key disease parameters in the animal model setting. The striking anti-inflammatory and modest anti-fibrotic remodelling effects of pinocembrin administration were likely linked to its ability to improve lung pathology and functional compliance.

## Supporting information

S1 Fig Representative Flash chromatography run of *Eucalyptus* extract showing the collected pinocembrin peak from 2.1 to 2.5 min. The pinocembrin peak was collected from multiple runs and pooled to provide sufficient material for testing in the sheep trial.
(PDF)

S2 Fig Quantitative analysis of the purity of pinocembrin isolated from *Eucalyptus* and used in the sheep trial. A, HPLC-PDA data of pinocembrin purified from a *Eucalyptus* extract using Flash chromatography and injected at a concentration of 1 mg ml^-1^. B, HPLC-PDA data of an authentic standard of pinocembrin purchased from Sigma-Aldrich and also injected at a concentration of 1 mg ml^-1^.
(PDF)

S1 Data (XLSX)
