# Supplementary material for: The efficacy and safety of pinocembrin in a sheep model of bleomycin-induced pulmonary fibrosis
Source: PLoS One. 2021 Dec 2;16(12):e0260719. doi: 10.1371/journal.pone.0260719 (PMC8638960; doi:10.1371/journal.pone.0260719)
Supplement: S1 Fig — The pinocembrin peak was collected from multiple runs and pooled to provide sufficient material for testing in the sheep trial. (PDF) [file pone.0260719.s001.pdf]

Method Name: PC 2020/Epp 5 min mtd
Run Name: Epp PC GRETALS_2020/2020-07-16_12-18-s2 1ml
Run Date: 202007-16 12:21

Column: Reveleris® C18-WP 12g	Solvent A: Water	UV Threshold: 0.05 AU	ELSD Threshold: N/A
Flow Rate: 30 mL/min	Solvent B: Acetonitrile	UV Sensitivity: High	ELSD Sensitivity: Low
Equilibration: 1.0 min	Solvent C: Empty	UV1 Wavelength: 288 nm	Collection: Collect None
Run Length: 5.0 min	Solvent D: Empty	UV2 Wavelength: N/A	Per-Vial Volume: 25 mL
Mode: Flash Liquid	Slope Detection: High	UV3 Wavelength: N/A	Non-Peak Volume: 25 mL

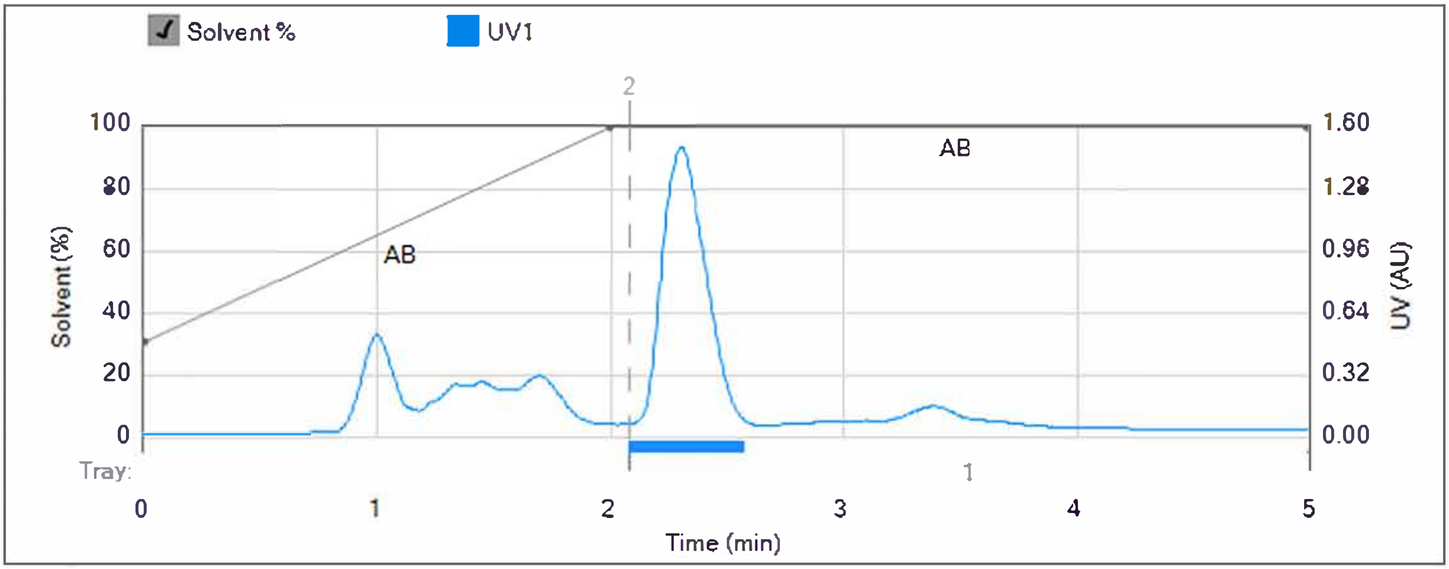

1 - 00A4

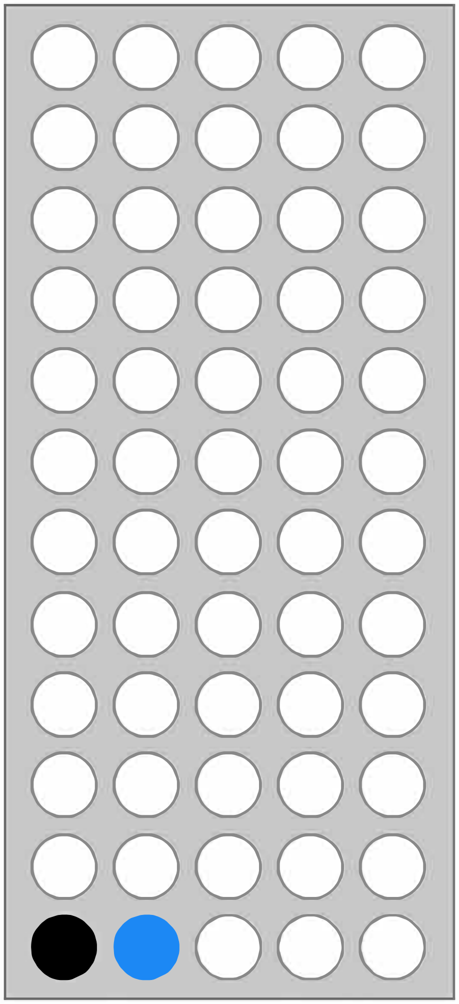

Gradient Table

	Min	Solvents	% 2nd
1	0.0	AB	30
2	2.0	AB	100
3	3.0	AB	100

Vial Mapping Table

Peak #	Start Tray:Vial	End Tray:Vial
1	1:2	1:2
