# Supplementary figures and images for: The efficacy and safety of pinocembrin in a sheep model of bleomycin-induced pulmonary fibrosis
Source: PLoS One. 2021 Dec 2;16(12):e0260719. doi: 10.1371/journal.pone.0260719 (PMC8638960; doi:10.1371/journal.pone.0260719)

A.

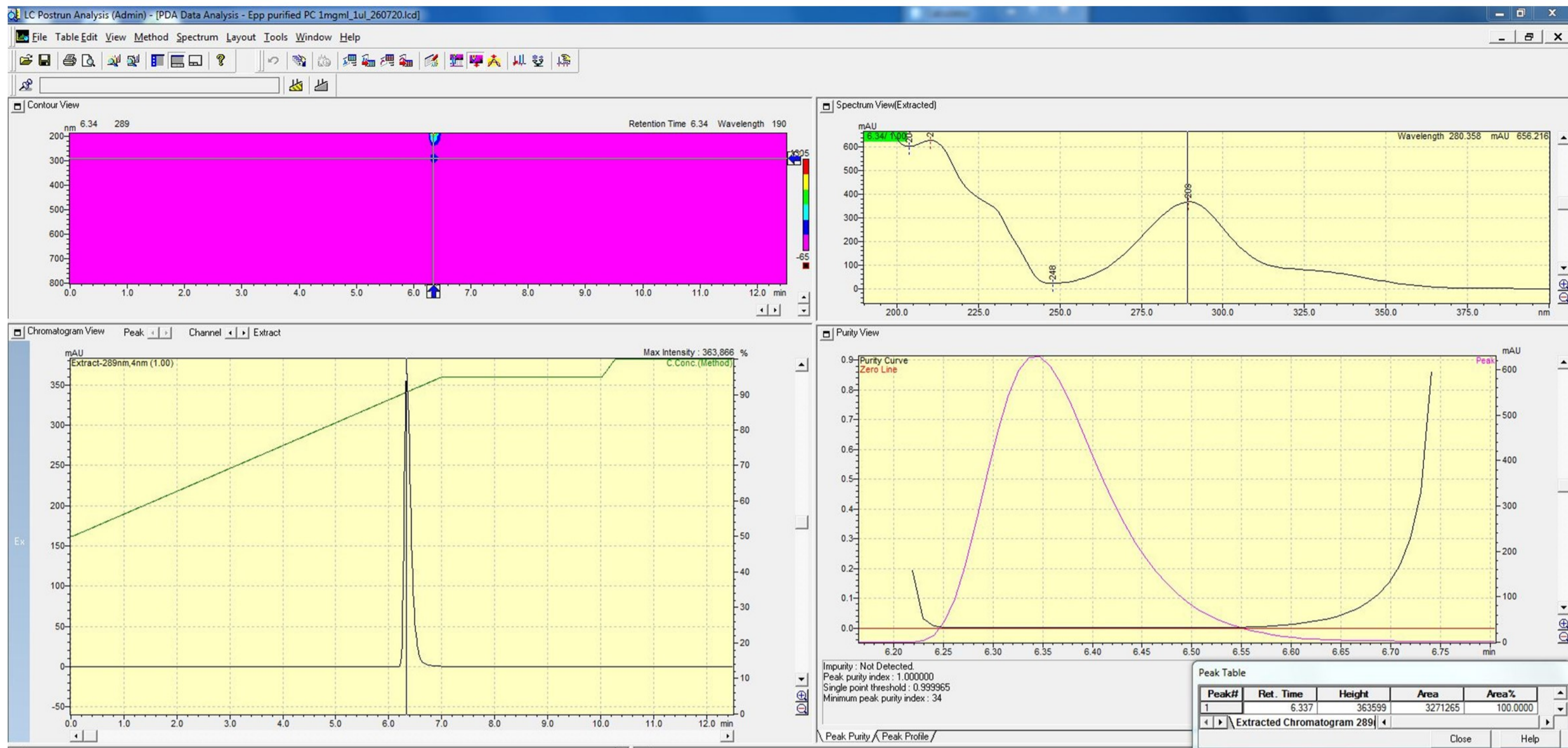

B.

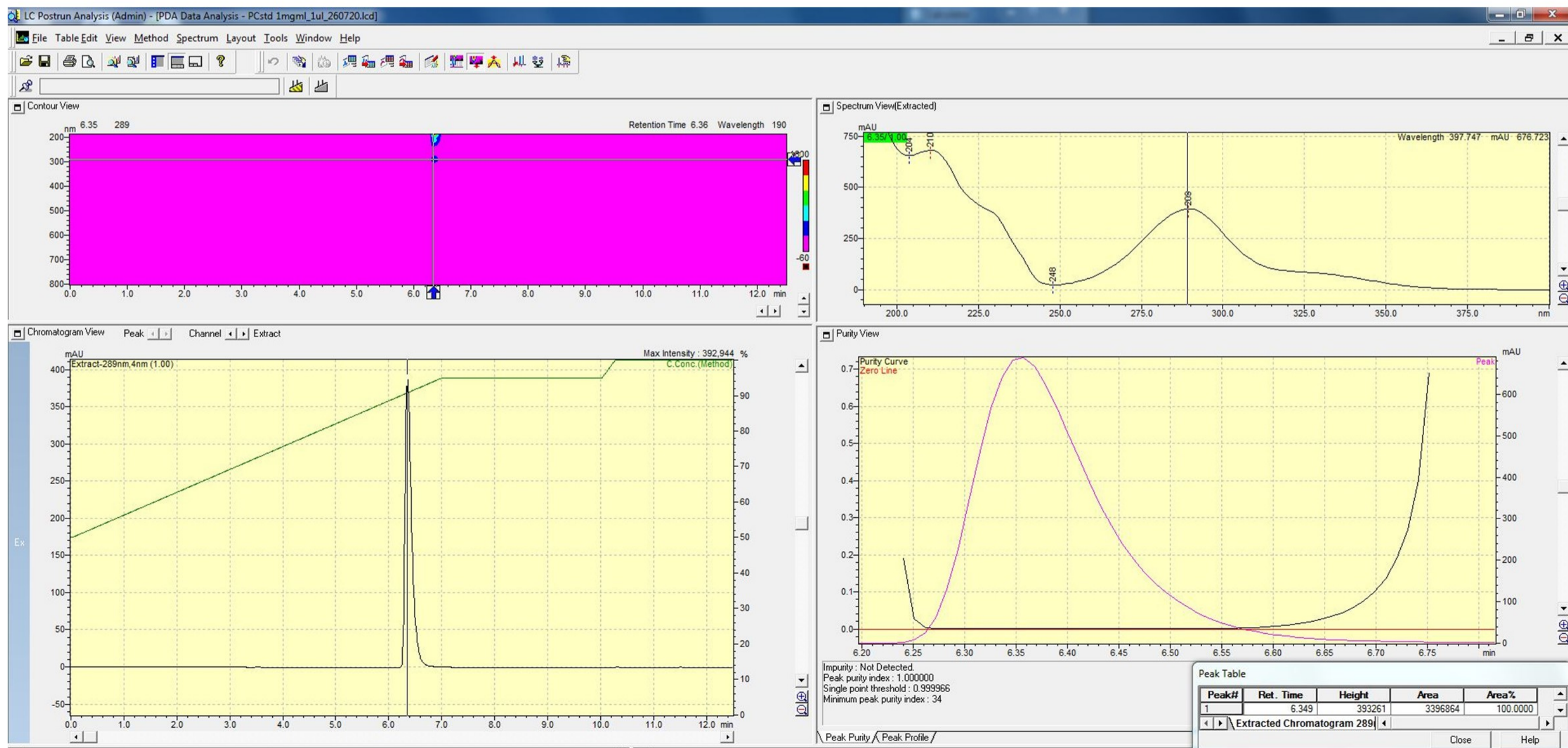

Supplement: S2 Fig — A, HPLC-PDA data of pinocembrin purified from a Eucalyptus extract using Flash chromatography and injected at a concentration of 1 mg ml-1. B, HPLC-PDA data of an authentic standard of pinocembrin purchased from Sigma-Aldrich and also injected at a concentration of 1 mg ml-1. (PDF) [file pone.0260719.s002.pdf]
